# A Comprehensive Review of Hass Avocado Clinical Trials, Observational Studies, and Biological Mechanisms

**DOI:** 10.3390/nu13124376

**Published:** 2021-12-07

**Authors:** Mark L. Dreher, Feon W. Cheng, Nikki A. Ford

**Affiliations:** 1 Nutrition Science Solutions, LLC, 900 S Rainbow Ranch Rd., Wimberley, TX 78676, USA; nss3@sbcglobal.net; 2 Avocado Nutrition Center, 25212 Marguerite Pkwy Ste. 250, Mission Viejo, CA 92692, USA; feon@hassavocadoboard.com

**Keywords:** Hass avocado, blood lipids, endothelial health, lipoprotein particle size, body weight, cognitive function, colonic microbiota, satiety, oleic acid, dietary fiber, lutein, carotenoids, energy density

## Abstract

This first comprehensive review of fresh Hass avocados includes 19 clinical trials, five observational studies, and biological mechanisms. We identified four primary avocado health effects: (1) reducing cardiovascular disease risk in healthy overweight or obese adults with dyslipidemia by lowering non-HDL-C profiles, triglycerides, LDL oxidation, small atherogenic LDL particles and promoting postprandial vascular endothelial health for better peripheral blood flow; (2) lowering the risk of being overweight or obese, supporting weight loss, and reducing visceral fat tissue in overweight or obese women; (3) improving cognitive function in older normal-weight adults and in young to middle age overweight or obese adults especially in frontal cortex executive function; and (4) stimulating improved colonic microbiota health in overweight or obese adults by promoting healthier microflora and fecal metabolites. We also identified a unique combination of four Hass avocado nutritional features that appear to be primarily responsible for these health effects: (1) a 6 to 1 unsaturated (rich in oleic acid) to saturated fat ratio similar to olive oil; (2) a source of multifunctional prebiotic and viscous fiber; (3) a relatively low energy density of 1.6 kcal/g (79% of edible Hass avocado weight consists of water and fiber with a creamy, smooth texture); and (4) its oleic acid and water emulsion increases carotenoid absorption from low-fat fruits and vegetables (e.g., salsa or salad) when consumed with avocados. They are also a good source of micronutrients and polyphenols, and are very low in sodium and available carbohydrates supporting secondary health and wellness benefits. Hass avocado health effects are best demonstrated when consumed in a healthy dietary plan such as the Mediterranean diet. More extensive and longer clinical trials are needed to further enhance our understanding of the Hass avocado’s health effects.

## 1. Introduction

Global dietary guidelines, such as the 2020–2025 Dietary Guidelines for Americans, call for making half of each meal fresh fruits and vegetables consumed in a healthy dietary plan such as the Mediterranean diet to promote healthy body weight, reduce chronic disease risk, and promote better health in all age groups [1]. Fresh fruit and non-starchy vegetables tend to have a very low to low energy density (ED), low sugar, sodium, and saturated fat content. They are often good sources of fiber, vitamins A, C, and K, magnesium and potassium, and phytochemicals such as polyphenols and carotenoids that collectively support health and wellness [1,2]. However, only 10–20% of the US population meets the dietary recommendations for fruit and vegetable intake, leading to an average healthy eating index score of about 60% of the optimum dietary quality score needed to protect against overweight, obesity, and chronic diseases that compromise the wellness of Americans and other global populations in all age groups [1,2].

Hass avocado (Persea americana) fruit makes up at least 90% of the avocados consumed in the US and most of the avocados consumed worldwide [3,4]. The Hass avocado has a creamy, smooth, edible fruit texture when ripe that is rich in oleic acid, fiber, micronutrients, and phytochemicals. The tree thrives in Mediterranean-type climates and is compatible with the Mediterranean diet [5,6]. A serving of fresh Hass avocado (50 g or 1/3 of a medium-sized fruit) contains 80 kcals, 3.4 g fiber (11% DV), 44.5 μg folate (10% DV), 0.73 mg pantothenic acid (15% DV), 85 μg copper (10% DV), 10.5 μg vitamin K (10% DV), 254 mg potassium (7.5% DV), and 4 mg of sodium (0.2% DV) [6,7]. Hass avocados have a relatively low energy density of 1.6 kcals/g (79% of weight consists of water and fiber), and a glycemic index and load of near zero due to a very low level of available carbohydrates comprised of 0.15 g sugar and 0.055 g of starch. One serving of Hass avocado contains no cholesterol, 1 g of saturated fatty acids (SFA, 5% DV), 4.9 g cis-monounsaturated fatty acid (MUFA), and 1 g polyunsaturated fatty acid (PUFA), with oleic acid as the predominant fatty acid at 4.5 g/serving. Also, each serving has 136 μg of lutein and zeaxanthin, 35 mg beta-sitosterol, and 95 mg total phenolics (gallic acid equivalents) [6,7]. Non-Hass varieties differ from Hass avocados in nutritional composition, especially in fatty acid levels [7,8]. National Health and Nutrition Examination Surveys (NHANES) from 2001–2012 showed that US Hass avocado adult consumers had a better nutrient intake, higher diet quality, and lower adiposity than non-consumers [4]. The average Hass avocado consumer eats 76 g/day or about 60% of one avocado, which leads to a significantly higher intake of fiber, cis-MUFA, vitamins E and C, folate, magnesium, potassium, total fruits and vegetables, and a lower intake of sodium compared to non-consumers.

There are no current comprehensive review articles or systematic reviews and/or meta-analyses that focus only on fresh Hass avocados, human health effects, and biological mechanisms. Previous narrative reviews, systematic reviews, and meta-analyses have included some non-Hass varieties in their analyses or are outdated. The objective of this comprehensive narrative review is to provide an overview of fresh Hass avocado clinical trials, observational studies, and biological mechanisms focusing on cardiovascular disease (CVD), weight management, cognitive function, and the colonic microbiota, and the primary nutritional features associated with these health effects.

## 2. Materials and Methods

This comprehensive narrative review of fresh Hass avocados includes published papers identified in a literature search of PubMed and Google Scholar between 1 June and 14 October 2021. Exclusion criteria included studies using non-Hass avocados and short-term blood lipid clinical trials lasting <four weeks in duration. Inclusion criteria include only confirmed Hass avocado randomized controlled trials (RCTs), including trials with a duration of ≥four weeks and acute postprandial trials, observational studies, systematic reviews, meta-analyses, and narrative review articles. The search criteria included: avocados, oleic acid, dietary fiber, energy density, lutein, carotenoids, blood lipids, LDL-oxidation, cardiovascular disease, glycemic control, inflammatory markers, flow-mediated dilation, vascular reactivity, body weight and composition measures, satiety, cognitive function, and colonic microbiota.

## 3. Results

This review includes 19 Hass avocado RCTs that met the acceptance criteria, but 7 RCTs were not included because they were either non-Hass avocados, were a combination of Hass and Sharwill avocados, or had a short duration of 1 to 3 weeks. Five Hass avocado observational studies were included. One older Hass avocado review was included [5], but two review articles were excluded because they primarily focused on avocado micronutrients and phytochemicals that were not specifically related to Hass avocados. Finally, three systematic reviews and/or meta-analyses that primarily assessed the effects of avocados on blood lipid profiles did not meet the inclusion criteria for this Hass avocado-specific paper [9,10,11]. Peou et al., in a 2016 meta-analysis, included ten unique trials, but only five were confirmed as Hass avocados, and two of the Hass avocado trials had durations of ≤2 weeks [9]. Caldas et al., in 2017, focused on avocado cardioprotective mechanisms with a qualitative systematic review of both Hass and non-Hass avocado effects on blood lipid profiles [10]. Mahmassani et al., in a 2018 systematic review and meta-analysis, assessed the effects of five Hass avocado trials and two trials, including either a non-Hass variety or a mix of Hass and Sharwill avocado varieties [11].

### 3.1. Cardiovascular Clinical Trials

#### 3.1.1. Individual Cardiovascular Clinical Trials with Hass Avocados

- Healthy Overweight or Obese Subjects

Three clinical trials leading to four publications in healthy overweight or obese subjects with dyslipidemia found that at least one avocado a day over 4 to 5 weeks significantly improved blood lipid profiles or reduced oxidized-LDL (ox-LDL) compared to control diets [12,13,14,15].

**Figure 1:**
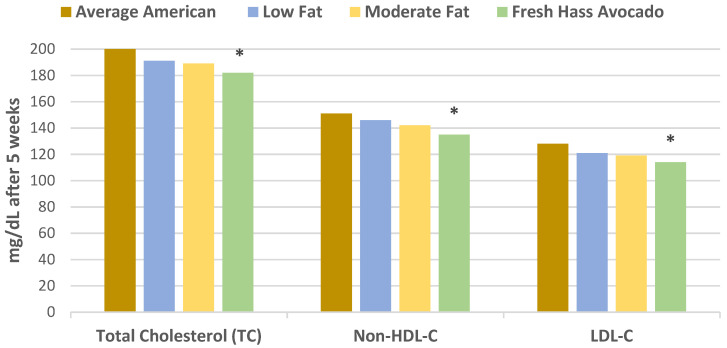
Compared to the average American diet, the Hass avocado diet significantly lowered TC by −18 mg/dL, non-HDL-C by −16 mg/dL, and LDL-C by −14 mg/dL (*p* < 0.005), more than the low-fat diet and the moderate-fat diet after 5 weeks (^∗^ *p* = 0.01) [12].

**Figure 2:**
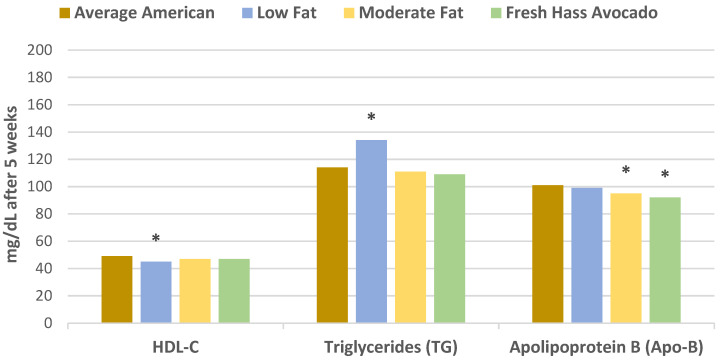
Compared to the average American diet: (1) the moderate fat and Hass avocado diets decreased HDL-C less than the low-fat diet (^∗^ *p* < 0.05); (2) the low-fat diet significantly increased TG by 18% (^∗^ *p* < 0.001), while there were small reductions in TG for the moderate fat and Hass avocado diets; and (3) both the Hass avocado and moderate fat diet significantly decreased Apo-B (^∗^ *p* < 0.0001) compared to the low-fat diet after 5 weeks [12].

**Figure 3:**
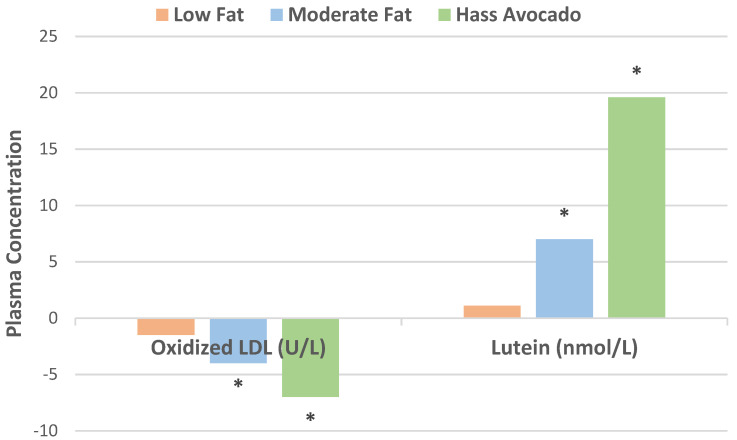
The Hass avocado diet significantly reduced ox-LDL plasma concentrations vs. low fat (^∗^ *p* = 0.03) and moderate-fat (^∗^ *p* = 0.05) and significantly increased lutein plasma concentrations vs. low-fat (^∗^ *p* = 0.0005) and moderate-fat (^∗^ *p* = 0.008) [13].

In a randomized crossover controlled feeding trial, overweight or obese adults (27 men and 18 women, mean age 45 years, mean BMI 28 kg/m^2^ and elevated LDL-C) consumed a run-in average American diet for 2 weeks before being randomized into one of three diets: (1) low-fat diet (about 59% carbohydrates, 24% fat, 7% SFA, 11% MUFA, 6% PUFA); (2) moderate-fat diet with 34% fat calories from oleic acid-rich sunflower and canola oils (49% energy from carbohydrates, 17% cis-MUFAs, 6% SFA, 9% PUFA); or (3) moderate-fat diet with one fresh Hass avocado per day (macronutrient profile same as the moderate-fat diet) for 5 weeks each [12]. Compared to the average American diet, the Hass avocado diet significantly reduced total cholesterol (TC), LDL-C, and non-HDL-C compared to the moderate fat and low-fat diets. Only the low-fat diet significantly increased triglycerides (TG) and very low density lipoprotein-cholesterol (VLDL-C). The data for these findings are summarized in Figure 1 and Figure 2. The Hass avocado diet was the only diet that significantly reduced the LDL: HDL ratio compared to the average American diet and significantly increased LDL particle size (less atherogenic) compared to the low-fat diet (*p* = 0.03). Stored frozen plasma samples from the previous clinical trial were subsequently analyzed for lutein and oxidation of small, dense LDL [13]. Compared with the average American diet, the Hass avocado diet significantly decreased plasma oxidized LDL and increased plasma lutein concentration (*p* = 0.0004 for both). The Hass avocado diet also significantly reduced ox-LDL and increased lutein compared to the moderate fat and low-fat diets (Figure 3). The lower ox-LDL level from the Hass avocado diet was significantly correlated with fewer small density LDL (sdLDL) particles (*r* = 0.32, *p* = 0.0002).

In a crossover RCT, Hispanic hypercholesterolemic adults (11 women and 2 men, mean age 56 years, mean BMI 29 kg/m^2^) were randomly assigned to one of two isocaloric high carbohydrate vegetarian diets: (1) an avocado free diet with 70% energy from carbohydrates and 20% energy from soybean and safflower oils; or (2) a Hass avocado diet with 60% energy from carbohydrates and 30% of energy from Hass avocados contributing 75% of the fat for 4 weeks [14]. Compared to the high carbohydrate avocado-free vegetarian diet, the high carbohydrate Hass avocados vegetarian diet reduced TC by −2% and LDL-C by −5.7% and had a 7% lower increase in TG and an 11% smaller decrease in HDL-C. Hass avocados appear to be most effective in lowering blood lipids in diets with up to about 50% energy from carbohydrates.

In a randomized trial, hypercholesterolemic Hispanic adults (14 women and 2 men, mean age 58 years, mean BMI 30 kg/m^2^) were randomly assigned to one of two isocaloric low saturated fat diets with either no avocados or a diet with at least one and a half Hass avocados per day for 4 weeks [15]. Compared to the low saturated fat diet without avocados, the Hass avocados enriched low saturated fat diet reduced TC by −31 mg/dL, LDL-C by −34 mg/dL, TG by −35 mg/dL, and increased HDL-C by 7.6 mg/dL.

- Overweight or Obese Type 2 Diabetic (T2D) Patients

Two Mexican clinical trials confirm that Hass avocados can be included in the American Diabetes Association (ADA) diet plan to manage CVD and T2D risk [16,17].

In a crossover RCT, T2D patients (mean age 56 years, mean BMI 28, elevated LDL-C) were randomly assigned to one of two isocaloric ADA diets: one diet with a Hass avocado and four teaspoons of olive oil (both with a 6 to 1 unsaturated to saturated fat ratio) or to a standard ADA diet for 4 weeks [16]. Each diet had similar reductions in non-HDL-C and blood glucose levels with no changes in HDL-C levels. Compared to the standard ADA diet, the Hass avocado and olive oil-enriched ADA diet reduced plasma TG by −13%.

In another crossover RCT, T2D patients (elevated LDL-C) were randomly assigned into one of two ADA type diets (50% carbohydrates, 20% proteins, and 30% fat rich in unsaturated fat): (1) avocado free control; or (2) Hass avocado contributing 75% of the fat for 4 weeks [17]. Both diets had similar reductions in fasting plasma glucose, glycosylated hemoglobin, insulin sensitivity, TC, LDL-C, and TG (*p* > 0.05), with no change in HDL-C from baseline levels.

- Normal Weight Subjects

One trial including normal-weight subjects with relatively healthy baseline overall lipid profiles showed only a trend toward lower TG with avocado intake, compared to an increasing trend for the control.

In a randomized controlled trial, healthy adults (mean age 63 years, normal BMI of 24 kg/m^2^, 63% women, near-optimal LDL-C, and relatively desirable HDL-C and TG) were randomly assigned to one of two diet groups with subjects instructed to consume one Hass avocado (treatment group) or one potato or one cup of chickpeas (control group) each day for 6 months [18]. There were no significant differences in TC, LDL-C or HDL-C, or LDL oxidative lag time between the avocado and the control diets. However, at six months, there was a trend for TG to decrease from baseline with the avocado diet by −7 mg/dL (*p* = 0.075) compared to an increase with the control diet of 9 mg/dL (*p* = 0.06). There were no significant changes in oxidative stress or inflammation measures over 6 months in either diet group. In post-hoc analyses, there was a significant increase in lutein serum levels from baseline at both three and six months of >25% (*p* = 0.001) in the avocado group.

#### 3.1.2. Acute Postprandial Clinical Trials of Cardiovascular Health Biomarkers

Four postprandial trials studied the effects of Hass avocados on endothelial health by peripheral arterial tonometry (PAT), flow-mediated dilation (FMD), or nitric oxide [19,20,21,22]. Two of these trials measured the effects of Hass avocados added to hamburger patty meals [19,20]. One trial compared an avocado sandwich with other meal options such as hamburgers, donuts, or fruit [21]. Another trial assessed the effects of the inclusion of avocado as a replacement for carbohydrates in a breakfast meal [22]. These trials showed significantly better endothelial health as measured by vasodilation in normal and overweight subjects for enriched avocado meals, compared to the control diets rich in saturated fat or available carbohydrates.

- Hass Avocado vs. Animal Fat (hamburger) and Other Foods

**Figure 4:**
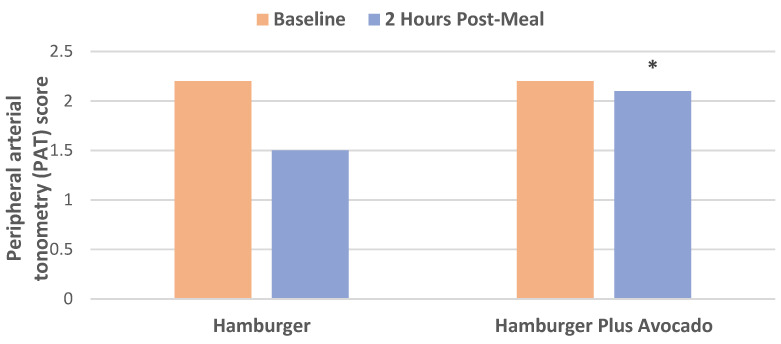
Postprandial 2 h after consuming hamburger plus half an avocado compared to plain hamburger improved PAT score compared to baseline (^∗^ *p* < 0.005) [19].

A crossover RCT investigated the postprandial effects of the addition of half a fresh avocado (68 g) to a 250 g (approx. ½ lb) hamburger patty on vasodilation and inflammation in eleven male subjects (mean age 25 years, and mean BMI 24 kg/m^2^) [19]. Vasodilation measurements by PAT over two hours showed that consuming one-half an avocado with a hamburger patty prevented the reduction in vasodilation associated with eating hamburgers alone (Figure 4). Also, the avocado and hamburger combination reduced systemic inflammation by: (1) significantly preserving I kappa B alpha activity, a well-recognized regulator of inflammation response, (131% vs. 58%, *p* = 0.03) compared to hamburger alone after 3 h; and (2) avocado plus the hamburger blocked a significant increase in serum IL-6 compared to the hamburger alone over 4 h. NHANES data found the average daily fresh avocado consumption by avocado consumers to be 76 g/day, higher than the avocado level used in this trial [4].

A crossover RCT investigated the postprandial effects of adding half a fresh Hass avocado to a 235 g hamburger patty in 14 patients with metabolic syndrome (80% men, mean age 36 years) [20]. Compared to the hamburger alone, the hamburger with half an avocado improved systemic nitric oxide levels by 65% and blocked lipid peroxidation levels by 60% over 4 h.

**Figure 5:**
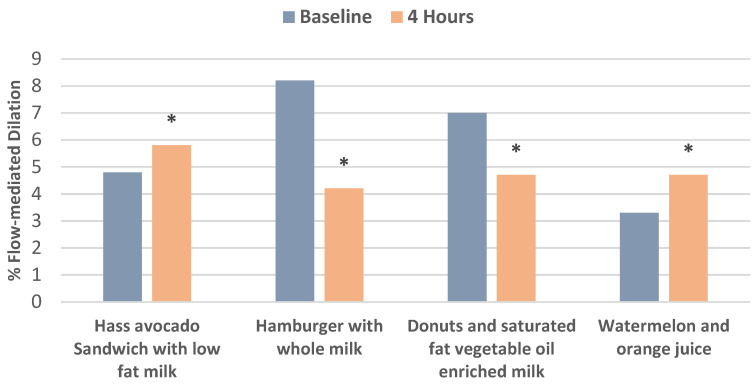
Isocaloric meals of a Hass avocado sandwich with low-fat milk and fruit consisting of watermelon and orange juice tended to increase flow-mediated dilation compared to significantly lower flow-mediated dilation (FMD) after hamburger and whole milk and donuts with saturated rich vegetable oil enriched milk after 4 h (^∗^ *p* < 0.05) [21].

A four-arm RCT in healthy young overweight men investigated postprandial effects on endothelial health after the isocaloric consumption of (1) a Hass avocado sandwich with low-fat milk; (2) hamburger plus whole milk; (3) donuts rich in saturated fat vegetable oil, and sugar; and (4) watermelon and orange juice, by measuring FMD compared to baseline over 4 h with 7 days between each food [21]. The Hass avocado sandwich, watermelon, and orange juice significantly improved FMD, compared to the poorer FMD measures after consuming the hamburger and donut meals (Figure 5).

- Hass Avocado vs. Carbohydrates

**Figure 6:**
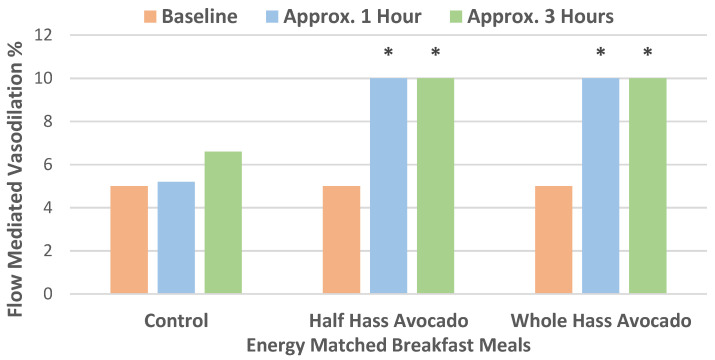
Postprandial flow-mediated vasodilation (FMD) percent response over 3 h after consuming each breakfast meal, with both Hass avocado meals significantly increasing FMD compared to the carbohydrate control meal (^∗^ *p* < 0.01) independent of dose [22].

In a crossover RCT, overweight/obese young to middle-aged adults were randomized into three energy-matched breakfast meals containing: (1) half a fresh Hass avocado and 78 g carbohydrates; (2) one whole fresh Hass avocado and 81 g carbohydrates; or (3) a control with no avocado and 120 g carbohydrates for assessment of postprandial FMD, glycemic control, and lipoprotein profiles [22]. FMD responses were significantly improved for the avocado meals compared to the high carbohydrate control meal (*p* < 0.01), independent of dose, as shown in Figure 6. The avocado meals significantly reduced glucose and insulin responses when compared to the carbohydrate control meal with a lower peak glucose concentration of 1.1 mmol/L (*p* < 0.0001), and peak insulin concentrations were lowered by 75 pmol/L (*p* < 0.005). There were no significant differences between the half and whole Hass avocado meals. Compared to the control diet, nuclear magnetic resonance analyses of lipids and lipoproteins measured at 2, 4, and 6 h found that a whole avocado breakfast significantly lowered concentrations of triglyceride-rich lipoproteins (*p* = 0.02) and increased concentrations of larger HDL particles (*p* < 0.05).

### 3.2. Body Weight Management

Four observational and five clinical studies evaluated the effects of Hass avocados on weight management or hunger and satiety [4,23,24,25,26,27,28,29,30,31,32]. Current dietary guidelines recommend the consumption of an adequate intake of a variety of fresh fruits and vegetables to help maintain and achieve a healthy weight through reducing eating rate, lowering the total dietary energy density (ED), and increasing fiber intake by displacing other foods and snacks that are moderate to high in ED and low in fiber [1,31]. All nine studies show that Hass avocados are consistent with dietary guidelines calling for an increased intake of fresh fruits and vegetables to support weight management, specifically by reducing the risk of being overweight or obese, or facilitating weight loss in reduced energy diets.

#### 3.2.1. Observation Studies

- National Health and Nutrition Examination Surveys (NHANES)

Two NHANES studies analyzing data between 2001 and 2012 showed that US adult consumers of Hass avocados had lower body weight, BMI, and waist circumferences than non-avocado consumers, by 3 kg, 1 kg/m ^2^, and 3–4 cm, respectively [4,23]. Also, Hass avocado consumers had a 33 % lower risk of becoming overweight or obese, a 32% lower risk of enlarged waist circumference, and a 50 % lower risk of metabolic syndrome than non-consumers [4,23].

- Australian Health Survey’s National Nutrition and Physical Activity Survey (NNPAS)

Analysis of the cross-sectional, nationally representative 2011–2012 NNPAS found when adjusted for covariates that greater avocado consumption was associated with lower body weight, BMI, and waist circumference [32]. The mean intake of avocado was 2.56 g per day, with 15.9% of Australians classified as avocado consumers. For each 50 g serving of avocado, researchers predicted avocado consumers would weigh six pounds less than non-consumers.

- Prospective Studies

Two large prospective studies on Hass avocado intake related to change in body weight found that daily intake of avocados helps protect against weight gain over time [24,25].

In 2015, Bertoia et al. examined the association between overall and specific fruit and vegetable intake and change in body weight in three large US prospective cohorts of 133,468 men and women over 24 years within multiple 4 year time spans and adjusted for differences in lifestyle factors, including dietary and physical activity changes [24]. Per daily serving, mean total fresh fruits were about twice as effective at reducing weight by 0.24 kg (−0.28, −0.20 kg) compared to total vegetables by 0.11 kg (−0.16, −0.06 kg) over four years. Weight changes over 4 years for specific fruits and vegetables are summarized in Table 1, showing that a daily serving of Hass avocados leads to a slight decrease in mean body weight by 0.21 kg over 4 years, protects against weight gain, and may contribute to modest weight loss, depending on the number of servings consumed.

**Figure 7:**
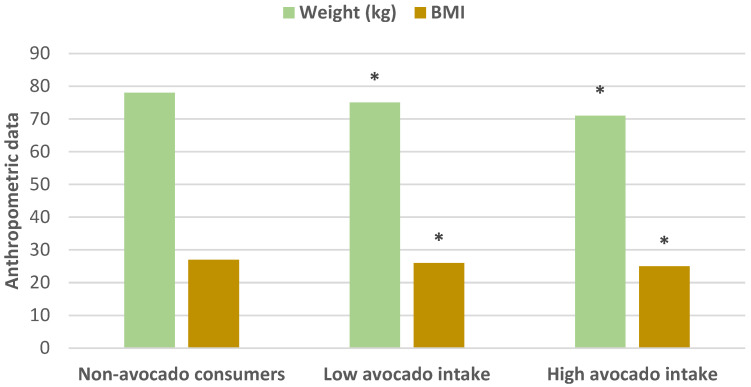
Effect of Hass avocado intake on body weight and BMI compared to non-consumers (^∗^ *p* < 0.0001) [25].

A large prospective study examined the effects of habitual Hass avocado intake on changes in weight and body mass index (BMI) in the Adventist Health Study (AHS-2) cohort (55,407 US and Canadian participants, mean age 56 years) over 5 years [25]. The study found that avocado consumers (≥32 g/day) who were normal weight at baseline increased weight at a slower rate of 0.26% compared to the non-consumer rate of 0.79%. High Hass avocado consumers were shown to have modest but significantly lower body weight and BMI than non-consumers (Figure 7).

#### 3.2.2. Clinical Trials

Two Hass avocado clinical trials support the daily intake of one daily avocado to help promote weight management when included in weight loss diets or in regular diets to lower visceral fat levels [26,27]. Three Hass avocado postprandial clinical trials showed that hunger was curbed, and meal satisfaction was increased over 3 to 6 h after the avocado was included at breakfast or lunch compared to the control meals [28,29,30].

- Weight Loss Diets

**Figure 8:**
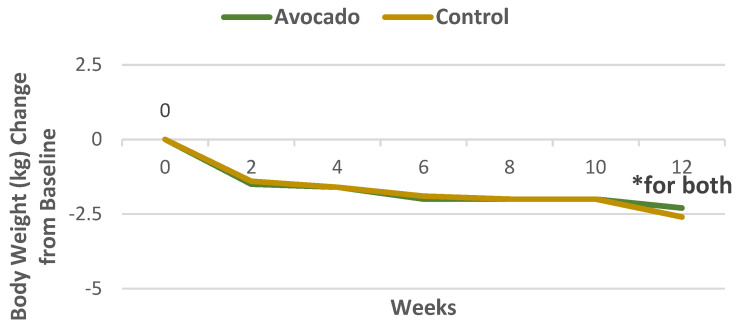
Bodyweight decreased for both avocado and control diets in overweight and obese subjects from baseline to 12 weeks (^∗^ *p <* 0.05) [26].

**Figure 9:**
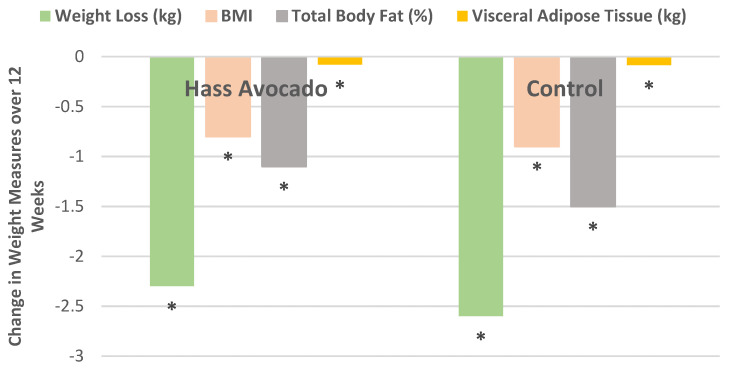
Change in body weight and composition measures from baseline to week 12 with both weight loss diets losing significantly (^∗^ *p* < 0.025 for all weight attributes) with no significant difference between the two diets [26].

A parallel RCT intervention randomly assigned 51 healthy overweight or obese adults (78% women, mean age 39 years, mean BMI 30, and mean body fat 40%) to isocaloric 500 kcal deficit diets with and without a daily Hass avocado for 12 weeks [26]. There were no statistically significant differences in change of body weight, BMI, total fat (%), or visceral fat (kg) between the two diets (Figure 8 and Figure 9). However, the avocado diet had a small but significantly higher satiety score by 0.26 compared to the control diet (*p* < 0.05).

- Regular Diets

A parallel RCT randomly assigned 105 adults (mean age 35 years, mean BMI 33) to an isocaloric daily meal with one Hass avocado or a control with similar foods without a Hass avocado for 12 weeks [27]. The consumption of a diet with a daily avocado altered abdominal fat distribution from baseline to 12 weeks in women but there were no improvements in body weight, insulin resistance (HOMA-IR), insulin sensitivity, or β-cell function in either men or women. The control diet had an unexpected significant reduction in subcutaneous abdominal adipose tissue (SAAT) by −55 g vs. an increase of 17 g for the avocado group (*p* = 0.017). Further, the control diet increased the visceral adipose tissue (VAT) to a SAAT ratio of 0.007 compared to a reduction of −0.011 for the avocado group (*p* = 0.024). In women, the avocado group had a greater reduction in VAT of −33 g compared to an increase of 1.6 g for the control group (*p* = 0.021), and the VAT to SAAT ratio was reduced by −0.01 for the avocado group compared to an increase of 0.01 for the control group (*p* = 0.001). Among the men, there were no significant changes in abdominal adiposity.

- Hunger, Appetite, and Satiety

A crossover RCT randomly assigned overweight or obese adults (52% women, mean age 38, mean BMI 29) to one of three isocaloric breakfast meals with approximately 640 kcals: (1) a low-fat control (76% carbohydrate energy, 14% fat energy, and 5 g fiber); (2) half a Hass avocado with breakfast (51% carbohydrate energy, 40% fat energy, 8.6 g fiber); and (3) a whole Hass avocado with breakfast (50% carbohydrate energy, 43% fat energy, and 13.1 g fiber). Subjective satiety and appetite scores were collected for visual analog scales, and satiety hormones were measured in the blood over 6 h [28]. Hunger was curbed after the whole avocado breakfast compared to the control breakfast by about 18% (*p* < 0.05). Meal satisfaction was increased after both avocado breakfasts compared to control by 25% (*p* < 0.0005 for both). Breakfast-related changes in how much subjects wanted to eat over 6 h showed a trend for greater hunger suppression with increasing amounts of avocado intake (*p* = 0.11). Peptide YY hormone, which functions to slow down the movement of food in the digestive tract, was significantly elevated after the whole avocado breakfast by 2 ½ fold within 30 min, to increase satiety and reduce hunger compared to the control breakfast. After the low-fat control breakfast, peptide YY accounted for only a minor role in satiety and hunger responses.

In a crossover RCT, healthy overweight and obese adults (62% women, mean age 41 years, and mean BMI 28) were fed a standardized breakfast (490 kcals) and randomly assigned to one of three lunches: (1) avocado free control (680 kcals); (2) ½ Hass avocado inclusive (680 kcal); or (3) ½ Hass avocado added (800 kcals) with postprandial satiety and subjective appetite scores and hormone blood measurements [29]. The ½ Hass avocado added to a lunch meal increased post-ingestive satiety for the following 3–5 h. Compared to the control lunch, a ½ avocado added to lunch increased meal satisfaction by 23% (*p* = 0.05) and decreased desire to eat by 28% for 5 h (*p* = 0.04). Also, adding half an avocado to the lunch meal attenuated the rise in postprandial blood insulin levels compared to the avocado-free lunch meal (*p* = 0.04). A posthoc analysis confirmed that postprandial gut hormone measurements, except for ghrelin, were consistent with the subjective appetite and satiety scores in the ½ avocado added lunch meals [30].

### 3.3. Cognitive Function

Two clinical trials [18,31] and one cross-sectional analysis [33] support the beneficial effects of avocado on cognitive function. These clinical trials found that avocado intake modestly improved cognitive function in older adults with normal weight after 6 months and in young to middle-aged overweight or obese adults after 3 months. Similar findings were observed in an analysis of NHANES 2011–2014 participants aged 60 or older comparing avocado consumers to non-consumers. Specifically, avocado consumers had significantly higher z-scores for Consortium to Establish a Registry for Alzheimer’s disease—immediate and delayed recall, and global cognition score, respectively (all *p* < 0.05 in adjusted models) [33]. More extensive and more prolonged trials are warranted to confirm these findings.

#### 3.3.1. Older Adults with Normal Weight

In a parallel RCT, healthy adults (63% women, mean age 63 years with relatively healthy blood lipids, normal BMI of 24 kg/m^2^) were randomly assigned to one of two isocaloric diet groups: (1) one daily Hass avocado (treatment group); or (2) one potato or cup of chickpeas/day (control group) added to diets [18]. Primary outcomes included serum lutein, macular pigment density (MPD), and a computerized battery of cognition tests designed to evaluate memory, processing speed, and attention assessed at baseline to six months. After 6 months, the avocado diet group increased serum lutein by 25% (*p* = 0.001) and the control diet increased lutein by 15% (*p* = 0.030) from baseline. However, only the avocado group significantly increased MPD from baseline (*p* = 0.001). Although both groups showed significant improvement in memory and spatial working memory (*p* ≤ 0.032) associated with better hippocampus function, only the avocado group improved frontal cortex executive function ability, including sustained attention (*p* = 0.033) and efficiency in managing problem situations (*p* = 0.036). Dietary guidance including one daily Hass avocado may help modestly improve or maintain cognitive function in older, normal-weight adults.

#### 3.3.2. Young to Middle-Aged Overweight and Obese Adults

**Figure 10:**
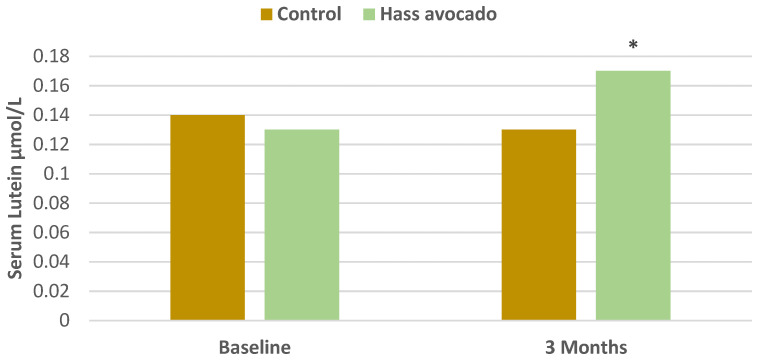
Change in serum lutein for Hass avocado group vs. baseline (^∗^ *p* < 0.001) [31].

**Figure 11:**
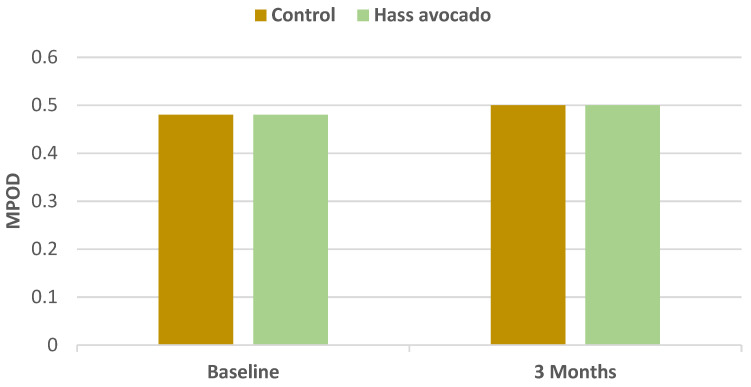
Change in macular pigment optical density (MPOD) for Hass avocado group vs. baseline and control group vs. baseline (*p* = 0.8) [31].

In a parallel RCT, young to middle-aged overweight and obese adults (57% women, mean age 34 years (20–45 years), mean BMI 32, mean body fat 37%) were randomly assigned to one of two isocaloric meals with either one Hass avocado or an avocado free control for 3 months [31]. Consistent with the *a priori* hypothesis, participants in the avocado group had significantly greater Flanker task accuracy by 2.2% (*p* = 0.1), congruent accuracy by 1.3% (*p* = 0.05), and incongruent accuracy by 3.0% (*p* = 0.01). This translates into improved attentional inhibition to suppress inappropriate information for a given context, which appears to be associated with a modestly improved frontal cortex executive function (Table 2). The avocado group also had a significant improvement in serum lutein status by 0.04 μmol/L (*p* ≤ 0.001) compared to the control group, but there were no significant changes in macular pigment optical density (MPOD) between the two groups, which was inconsistent with the second *a priori* hypothesis (Figure 10 and Figure 11). Neither serum lutein concentrations nor MPOD correlated with significantly improved working memory performance or neuroelectric changes in either group. Dietary guidance, including one daily Hass avocado, may help modestly improve executive function in overweight or obese young and middle-aged adults, which is important because excess adiposity can increase the risk for cognitive impairment.

### 3.4. Colonic Microbiota

Two clinical trials provide initial insights into how Hass avocados can improve the symbiotic environment of colonic microbiota and metabolites in overweight and obese adults on both regular and weight-loss diets over 12 weeks [26,34].

#### 3.4.1. Regular Diets

**Figure 12:**
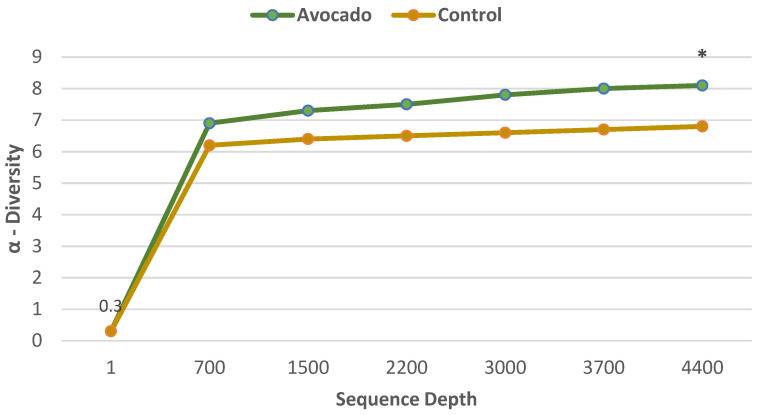
Effect of diet group on α diversity in overweight and obese adults (avocado group vs. control group (^∗^ *p-trend* = 0.02) [34].

**Figure 13:**
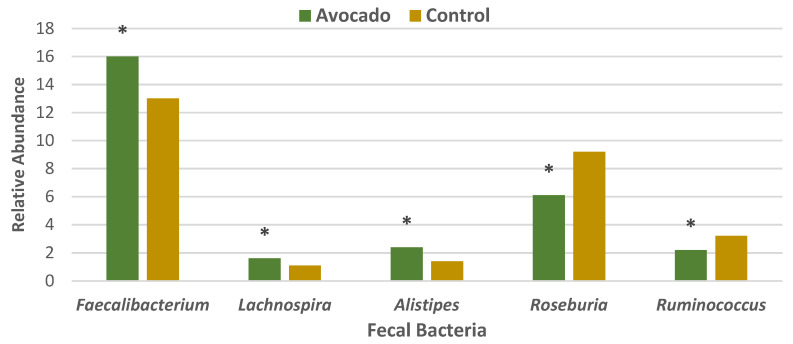
Analysis of the relative abundance of fecal bacteria with significant changes between dietary groups of overweight and obese adults (^∗^ *p* < 0.01 for all) [34].

**Figure 14:**
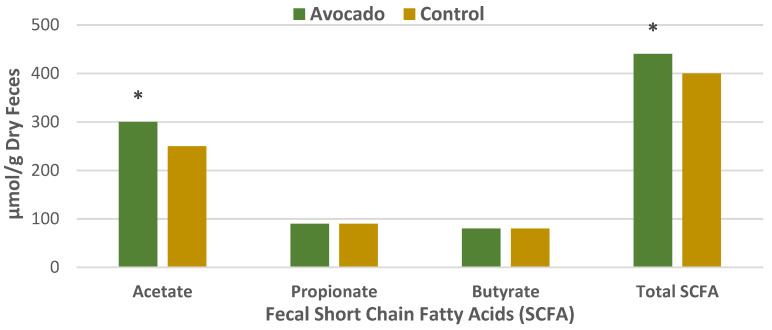
Hass avocado enriched diets significantly increased acetate concentrations ^∗^ *p* = 0.02 and directionally increased total short-chain fatty acids (SCFAs) vs. the control group in overweight and obese adults [34].

**Figure 15:**
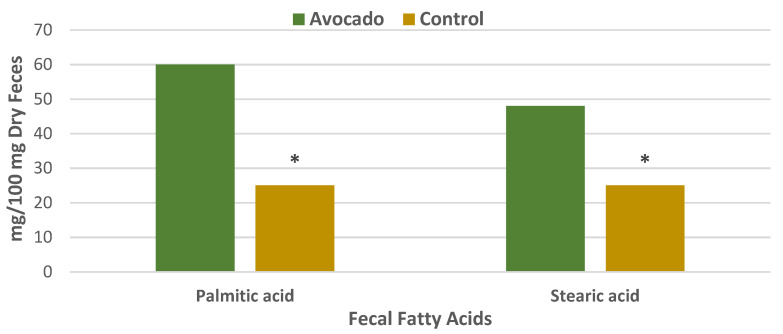
Hass avocado enriched diets significantly increased fecal concentrations of palmitic and stearic acids (^∗^ *p* < 0.05)) compared to the control diet in overweight and obese adults [34].

**Figure 16:**
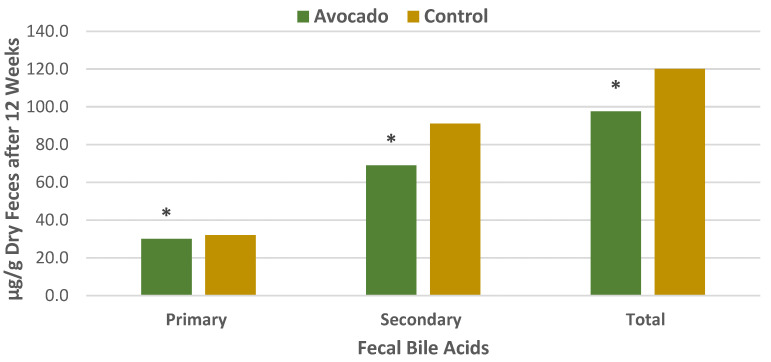
Hass avocado enriched diets tend to lower fecal primary, secondary, and total bile acid concentrations (^∗^ *p* < 0.1) vs. control diet in overweight and obese adults [34].

A parallel RCT was conducted in 157 overweight and obese adults (64% women, mean age 35 years, mean BMI 33, energy intake 1970 ± 289 kcals/day) who were randomly assigned to one of two isocaloric diets: (1) the avocado free control; or (2) one Hass avocado/day diet for 12 weeks [34]. Primary outcomes were fecal microbiota, fecal metabolites, and other health markers. The fecal microbiota was assessed with 16S ribosomal RNA gene sequencing, and analyses were made of fecal fatty acid and bile acid concentrations to assess bivariate correlations conducted between fecal microbiota, fecal metabolites, and health measures. The avocado group significantly increased α-diversity compared to the control group (Figure 12), and β-diversity tended to be greater for the avocado group than for the control (*p* = 0.09). At the genus level, the relative levels of *Faecalibacterium*, *Lachnospira*, and *Alistipes* were increased, and *Roseburia* and *Ruminococcus* were decreased compared to the control group (Figure 13). The avocado group had higher fecal acetate (*p* = 0.02) with a trend for higher fecal total short-chain fatty acids (SCFA) (Figure 14) and greater fecal stearic acid and palmitic acid concentrations compared to the control group, which appears to be associated with lower metabolizable energy (Figure 15). The avocado group tended to lower fecal primary, secondary, and total bile acid concentrations compared to control diets (Figure 16). This trial provides insights into Hass avocado’s beneficial effects on the colonic microbiota leading to improved metabolic health in at-risk overweight and obese adults.

#### 3.4.2. Weight-Loss Diets

A parallel RCT intervention randomized 51 healthy overweight or obese adults (78% women, mean age 39 years, mean BMI 30, and mean body fat 40%) into one of two isocaloric 500 kcal deficit diets, either with one daily Hass avocado or an avocado free control for 12 weeks [26]. Primary outcomes of this trial phase were fecal bacterial DNA sequencing and 16S rRNA gene identification with amplification. Although both diet groups lost similar body weight, the avocado group trended toward increased *Firmicutes* (*p* = 0.069) and no change in *Bacteroidetes*. In contrast, the control group trended toward decreased *Firmicutes* (*p* = 0.08) and increased *Bacteroidetes* (*p* = 0.05). The avocado group showed a decrease in *Bacteroides* and a trend toward increased *Prevotellaceae*, for a shift in microbiota to one more adapted for plant-based fiber and fat, compared to the control group that shifted microbiota to be more adapted to animal protein and fat intake. The relative proportion of bacterial phyla (*Firmicutes* and *Bacteroidetes*), family (*Bacteroidaceae* and *Erysipelotrichaceae*), and genus (*Bacteroides*, *Clostridium*, *Methanosphaera*, and *Candidatus Soleaferrea*) were significantly altered in the avocado group compared with the control group, but no significant changes in α- or β-diversity were observed. Also, there were trends toward a decrease in systemic inflammatory factors IL-1β by −0.13 pg/mL (*p* = 0.07) and C-reactive protein (CRP) by −7.2 μg/mL (*p* = 0.074) in the avocado group relative to the control. There was an increase in several taxa, which were significantly correlated with improved body weight and serum metabolic health biomarkers.

### 3.5. Carotenoid Bioavailability

Although dietary lipids are an important factor for carotenoid bioavailability, most fruits and vegetables are low in lipids, with lipid-rich avocado being an exception [35,36]. Two papers examined the effects of avocados and carotenoid bioavailability [35,36]. In the first trial of 11 healthy adults, researchers evaluated the effect of avocados consumed with salsa on the absorption of lycopene and β-carotene. Circulating levels of lycopene (4.4×) and β-carotene (2.6×) increased, compared to blood levels after the avocado-free salsa meals (*p <* 0.003). In the same publication, researchers compared eating avocados with a salad enhanced α-carotene (7.2×), β-carotene (15.3×), and lutein (5.1×) absorption to blood levels for meals without the avocado (*p* < 0.01). In a second trial, 12 healthy subjects consumed carotene-rich tomato sauce or carrots with avocado, which enhanced the concentration of circulating vitamin A compared to blood levels when no avocado was consumed.

## 4. Discussion

**Figure 17:**
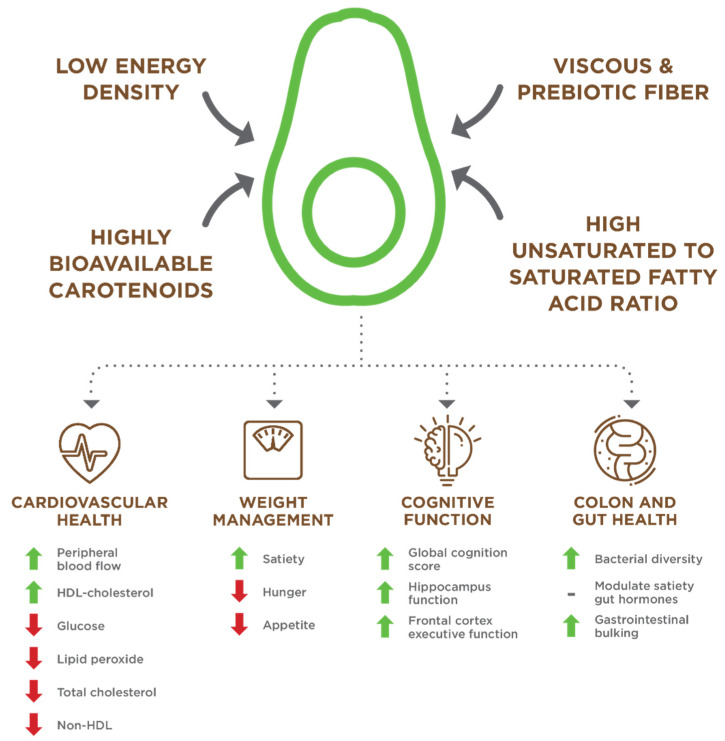
A combination of avocado nutrients and attributes contributes to the identified health benefits.

This study presents the first comprehensive review of Hass avocado clinical trials, observational studies, and biological mechanisms. We found that the Hass avocado has four major health benefits (Figure 17): (1) cardiovascular health [12,13,14,15,16,17,18,19,20,21,22]; (2), weight control [23,24,25,26,27,28,29,30]; (3) cognitive health [18,31,33]; and (4) colonic microbiota health [26,34]. We also found that four nutritional features were primarily responsible for all the major avocado health benefits: (1) high unsaturated to saturated fat ratio [7]; (2) viscous and prebiotic fiber [2,7]; (3) a relatively low energy density of 1.6 kcal/g [7]; and (4) high carotenoid bioavailability [35,36]. Avocados are also a unique nutrient/phytochemical dense fruit with minerals, vitamins, β-sitosterol, and polyphenols that provide additional secondary health benefits in promoting overall health and wellness to varying degrees. Still, more research is needed to understand their specific health effects better.

### 4.1. Biological Mechanisms

#### 4.1.1. High Unsaturated to Saturated Fatty Acid Ratio

Diets including avocados have a higher unsaturated to saturated fat ratio, which helps improve cardiovascular health, weight control, and cognitive function. For cardiovascular health, avocados are consistent with the American Heart Association’s Presidential Advisory Report recommending a shift from saturated to unsaturated fatty acids such as cis-MUFAs to promote healthier blood lipid profiles, including lowering LDL-C, a major cause of atherosclerosis [37]. Also, the avocados’ fat ratio helps improve vascular endothelial function by maintaining healthy postprandial FMD blood flow. The fat profile maintains low circulatory non-esterified fatty acids to optimize insulin sensitivity. This is critical as vascular endothelial cells depend on insulin activity for proper function. Also, the fatty acid profile likely reduces the risk of LDL oxidation, thus protecting against atherosclerosis [13,38,39,40,41,42,43]. For weight control, higher oleic acid intake leads to lower fat storage compared to long-chain saturated fats, especially when consumed in a moderate refined carbohydrate diet [44,45]. The mechanisms include converting oleic acid to oleoylethanolamide in the intestine and liver, which helps induce satiety and increase energy expenditure by stimulating a cascade of biochemical activities [46,47,48]. Oleic acid-rich diets improve cognitive function compared to low fat and high SFA diets [49,50,51,52,53,54,55]. Avocados’ healthy fatty acid ratio helps improve cerebral cortex blood flow, compared to long-chain saturated fats, to help assure the critical delivery of oxygen to the brain to match the increased needs of activated neurons [56,57].

#### 4.1.2. Viscous and Prebiotic

Diets with avocados contain a good source of viscous and prebiotic fiber, which help improve all four identified avocado health benefits. For cardiovascular health, avocados are consistent with the Third Report of the National Cholesterol Education Program Expert Panel and meta-analyses of RCTs, which showed that one gram of viscous pectin could significantly lower TC by −2.7 mg/dL and LDL-C by −2.1 mg/dL [58,59]. Pectin also slows the progression of intima-media plaque deposits in the common carotid arteries [60] and 4 g of fruit fiber daily lowers the risk of coronary heart disease by 8% [61]. The major mechanism for pectin and other fiber components is to increase viscosity in the ileal region of the small intestine, leading to reduced efficiency of saturated fat and cholesterol absorption and bile acid reabsorption, thus increasing the uptake of circulatory LDL-C by the liver to reduce the circulatory LDL-C load. Fruit fiber fermentation in the microbiota of SCFAs also reduces hepatic fatty acid synthesis to help lower LDL-C formation [62,63]. For weight control, numerous studies show that adequate fiber intake reduces body weight, waist circumference, and visceral fat, especially in women, compared to lower fiber diets [64,65,66,67,68,69,70]. The main mechanisms for increased fruit fiber intake and weight control are increases in gastrointestinal bulking volume that promote satiety hormones and reduce macronutrient bioavailability (e.g., reduced metabolizable energy), and promoting healthier microbiota metabolites and microflora that support a more metabolically lean phenotype [2,4,25,68,71,72]. For cognitive function, daily intake of a prebiotic type fiber of at least 5–10 g/day can help restore or maintain colonic microbiota homeostasis to reduce systemic inflammation and improve insulin sensitivity, which helps optimize the gut-brain axis for better hippocampal and frontal cortex function [73,74,75,76,77]. In young adults, avocado intake improved cognitive functions such as increased processing speed, sustained attention, and working memory in midlife [78]. For colonic microbiota health, adequate intake of fiber, especially prebiotics, can generate metabolically active SCFAs [74,75]. Fruits are among the best fiber sources for improving colonic microbiota health. As they ripen, their semi-hydrated cell wall fiber components, including pectin, hemicellulose, and cellulose, become progressively disassembled, and with eating and digestion, their fiber components become highly accessible to the colonic microbiome for fermentation [2,7]. Overall, fruit fiber acts as a prebiotic to help re-balance the colonic microbiota towards a higher anti-inflammatory profile by increasing the *Bacteroidetes*/*Firmicutes* ratio, increasing microflora diversity, optimizing colonic mucosal barrier, and lowering levels of primary and secondary bile acids, which are important for maintaining overall health [2,26,34].

#### 4.1.3. Low Energy Density (ED)

For weight control, avocados with an ED of 1.6 kcal/g (79% by weight of water and fiber) support better weight management and improve body composition [23,24,25,26,27,28,29,30,31,32]. Most fresh fruits have a high bulk volume and low energy density (ED), ranging from 0.3 to 1.6 kcal/g [2,5,6,7]. Humans tend to have relatively low energy regulatory sensitivity to food or meals with an ED greater than 1.75 kcal/g, leading to a positive energy balance and thus to an increased risk of weight gain [79]. According to NHANES analyses, the estimated US mean daily ED is 1.9 kcal/g, consistent with Western diets low in fruit and vegetable intake resulting in a higher risk of being overweight or obese over time [80]. A longitudinal study in young overweight women consuming higher ED diets ≥ 1.85 kcals/g showed an average weight gain of 6.4 kg over six years [81].

#### 4.1.4. Highly Bioavailable Carotenoids

Avocados are a unique, oleic acid-rich fruit that provides a highly bioavailable source of lutein, and the lipid boosts carotenoid absorption from co-consumed fruit and vegetables [35,36]. This nutritional feature supports cardiovascular health and cognitive function. For cardiovascular health, highly bioavailable lutein from avocados helps protect LDL from oxidation, decreasing the uptake of macrophages, and helps protect against atherosclerosis pathogenesis [82,83]. Observational studies show that higher lutein intake and blood levels are moderately associated with lowered coronary heart disease and stroke risk [84]. Combinations of lutein and lycopene are twice as effective at reducing carotid artery intima-media thickness than lutein alone [85], which may be achieved by consuming avocados with salsa or other tomato-based foods [35,36]. This can significantly lower mean LDL-C, increase FMD, and decrease systolic blood pressure [86]. For cognitive function, a higher intake of carotenoid-rich fruit and vegetables is generally associated with enhanced cognitive function in younger and older adults [87,88]. Several RCTs of lutein and zeaxanthin supplements after 12 months show improved visual episodic memory performance in young and middle-aged adults, and improved inhibition and attention performance in middle-aged and older adults compared to controls [89]. Importantly, avocado lutein is more highly bioavailable than that of supplements or low-fat fruits. Lutein and zeaxanthin can cross the blood-brain barrier and direct their antioxidant and anti-inflammatory functions to act directly on the brain structure and function with selective distribution to the prefrontal cortex, visual cortex, hippocampus, and central retina, as measured by macular pigment optical density [87,88,89,90,91,92]. The concentration of lutein in the brain is higher in the prefrontal cortex than other regions and positively correlated with the gray matter volume in the parahippocampal gyrus, suggesting that lutein plays a role in memory and inhibition functions [18,31,33,90,91,92].

### 4.2. Strengths and Limitations

Our review has several strengths and limitations. The strengths are that it: (1) identifies four primary Hass avocado health effects and their linkage to a unique combination of four primary nutritional features responsible for these benefits; (2) summarizes all the research in a short overview, with numerous figures and tables to help readers get a clear and complete assessment of Hass avocado research to date; (3) connects the existing Hass avocado research and primary nutritional features to previous research from major reports on dietary recommendations for reducing CVD risk, systematic reviews and meta-analyses, and clinical trials; and (4) calls for and identifies additional areas for future research and other study design gaps. The limitations are that: (1) many of the clinical trials were relatively short in duration using a small number of subjects; and (2) the clinical trials or observational studies were not scored for quality or assessed by statistical analysis of mean effects or heterogeneity supporting each health effect.

## 5. Conclusions

Clinical trials and observational studies have identified four primary Hass avocado health effects which promote: (1) cardiovascular health by improving blood lipid profiles and acute endothelial blood flow; (2) healthier weight and body composition; (3) better cognitive function, especially in areas of executive function; and (4) colonic microbiota health and related cardiometabolic and brain benefits. These health effects are primarily due to the Hass avocado’s unique combination of four nutritional features: (1) high oleic acid to SFA ratio; (2) multifunctional prebiotic and viscous fiber; (3) relatively low energy density; and (4) uniquely high bioavailable lutein along with other carotenoids, especially when avocados are consumed with other fruits and vegetables, i.e., in salads or salsa. Hass avocados are also micronutrient dense (e.g., 10% or more of the daily value for folate, vitamin K, pantothenic acid, and copper), very low in sodium and sugar, and contain polyphenols to further support secondary health and wellness benefits. Hass avocados are compatible with healthy dietary regimens such as the Mediterranean diet. Larger and longer clinical trials are needed to better understand the effects of Hass avocados on health, especially in glycemic and insulinemic control in normal and T2D subjects.

## Figures and Tables

**Table 1:** Change in body weight per daily serving of fruit and vegetables over 4 years in US men and women [24].

Fresh Fruit and Vegetables	Pooled 4 Year Weight Change (kg) per Serving (95% CI)
Blueberries	−0.63 (−0.76, −0.50)
Prunes	−0.58 (−1.04, −0.13)
Apples or Pears	−0.56 (−0.74, −0.27)
Strawberries	−0.39 (−0.64, −0.01)
Grapes	−0.32 (−0.45, −0.18)
Hass Avocados	−0.21 (−0.59, 0.17)
Grapefruit	−0.21 (−0.29, −0.12)
Melon	−0.13 (−0.41, 0.15)
Bananas	−0.10 (−0.22, 0.02)
Oranges	−0.07 (−0.17, 0.04)
Cauliflower	−0.62 (−1.03, −0.21)
String beans	−0.44 (−0.73, −0.15)
Peppers	−0.35 (−0.52, −0.18)
Broccoli	−0.39 (−0.34, −0.20)
Greeny leafy vegatables	−0.24 (−0.38, −0.10)
Carrots	−0.19 (−0.23, 0.15)
Beans	−0.18 (−0.36, −0.005)
Mixed vegetables	−0.15 (−0.31, 0.01)
Cabbage	0.18 (−0.10, 0.45)
Potatoes	0.34 (0.09, 0.59)
Peas	0.59 (0.17, 0.86)
Corn	0.93 (0.43, 1.43)

**Table 2:** Effect of Hass avocado on Flanker test components (* *p* = 0.05 and ** *p* = 0.01 for baseline vs. post testing) [31].

Flanker Test	Baseline		Post-Testing	
	Control Diet	Avocado Diet	Control Diet	Avocado Diet
Overall accuracy %	93.5 (4.7)	93.4 (5.3)	92.5 (5.9)	95.6 (3.5) **
Congruent accuracy %	97.2 (3.5)	96.5 (5.3)	95.7 (4.9	97.8 (2.9) *
Incongruent accuracy %	89.4 (7.6)	90.4 (6.3)	89.2(8.2)	93.4 (4.7) **
